# Understanding retort processing: A review

**DOI:** 10.1002/fsn3.3912

**Published:** 2023-12-27

**Authors:** Paulina Simoneth Jimenez, Sneh Punia Bangar, Mathew Suffern, William Scott Whiteside

**Affiliations:** ^1^ Department of Food, Nutrition and Packaging Sciences Clemson University Clemson South Carolina USA

**Keywords:** food preservation, retort technology, thermal processing

## Abstract

Retort processing is a food preservation technique to address the challenge posed by *Clostridium botulinum* for commercial sterility of a food product to get microbiologically safe and stable products by heating. This review aims to explore the journey of retort processing, starting from its early use in single‐batch canned foods and progressing to its contemporary applications with different types of containers and heating mediums. Additionally, it will delve into the adaptability of retort equipment, including its ability to operate in stationary and various agitation states, as well as its flexibility in processing speed for both single‐batch and continuous operations.

## Graphical abstract

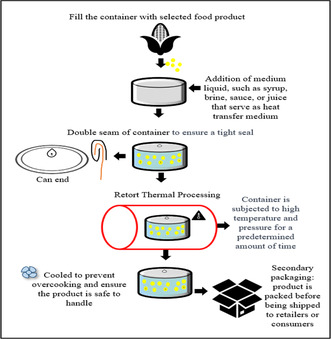

## 1 INTRODUCTION

Food contamination by microorganisms is a significant public health concern, where fungi cause deterioration and bacteria foodborne illnesses (Clark et al., 2014). Spores, such as those from *Clostridium thermosaccolyaticum*, *Bacillus* spp., and *Clostridium botulinum*, can also pose health risks as they can often be highly heat‐resistant and thrive under anaerobic conditions (Awuah, Ramaswamy, & Economides, 2007). Foodborne illnesses affect billions of people each year and impose a significant burden on public health globally (Seboka et al., 2023). To prevent this, it is crucial to implement procedures like sterilization and pasteurization to guarantee food safety.

Thermal processing is a widely used technique for preserving food and has gained increasing interest in recent years due to the demand for longer‐lasting high‐quality food (Singh et al., 2018). Heat treatment is a commonly used thermal processing technology in the food industry because it is a safe, chemical‐free, and cost‐effective method for producing cooked aromas and flavors while extending shelf life. The primary aim of thermal treatment is to destroy hazardous contaminants, including *C. botulinum*, which can also be prevented in food products by monitoring pH levels (≤4.6) and water activity (≤0.85). Sterilization is necessary to ensure the absence of bacteria under living conditions, but for commercial operations, extreme temperature exposure significantly reduces food quality. Instead, foods are treated through a process known as “commercial sterility” (Awuah, Ramaswamy, & Economides, 2007). The history of thermal processing dates to the early 19th century, with the development of canning by Appert, and improved by Peter Durand years later with the invention of the tin can. Static retorts, heated with steam as the medium, have been used in industrial purposes for a long time, and new other versions were created such as water, steam, air, water cascades, and water spray systems. Improvements in the thermal retort process include agitation and changes in processing speed (Tucker & Featherstone, 2021). Numerous studies have been conducted to determine optimal methods for operating retorts to different food products. This review will review methods and technologies relevant to the retort process.

## 2 SUMMARY OF RETORT PROCESSING

### 2.1 Retort processing

**FIGURE 1:**
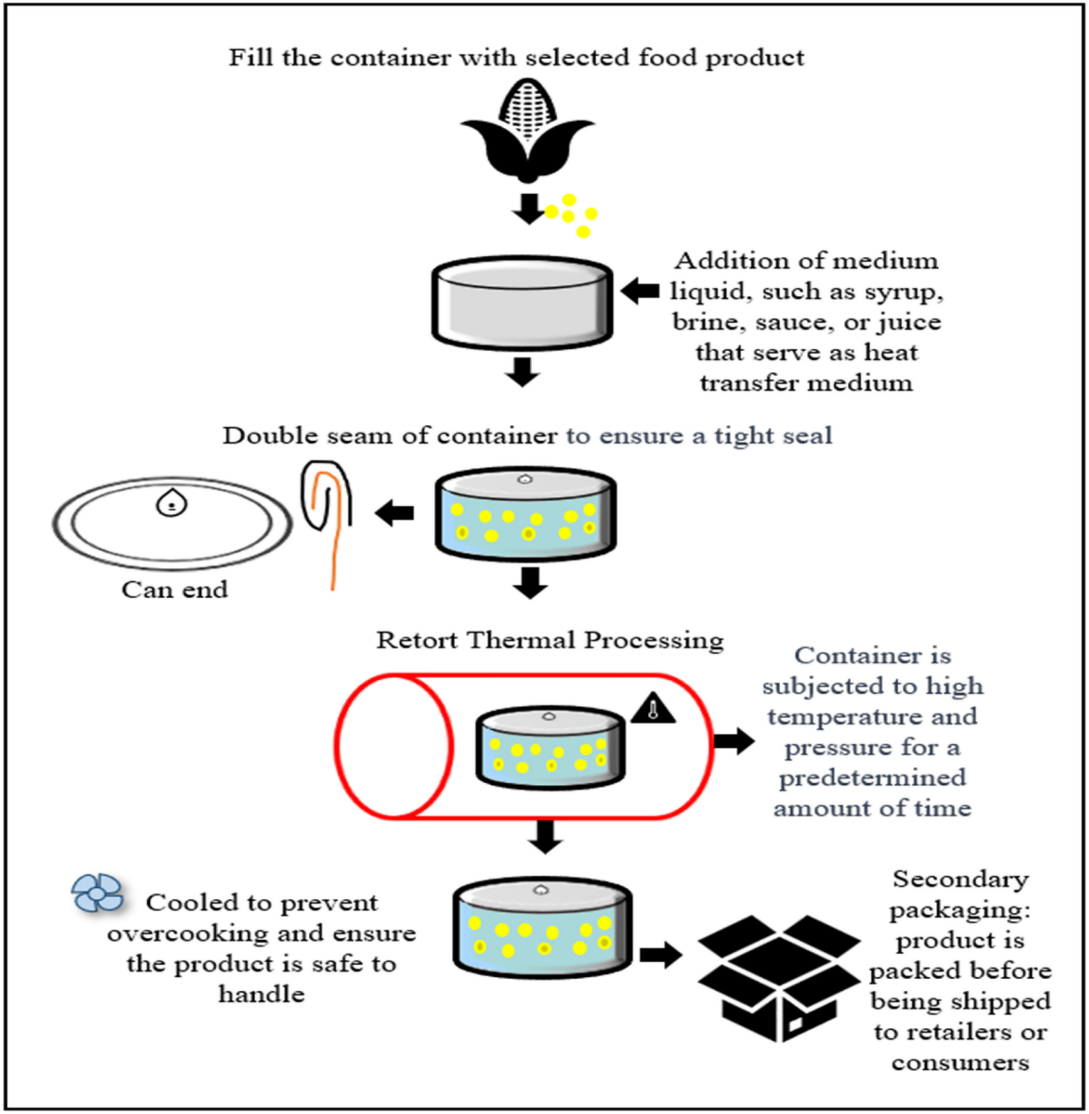
Standard commercial retort canning process.

The commercial sterilization method involves using heat to raise the temperature of the containers in a commercial closed vessel known as a retort or autoclave; principles are shown in Figure 1. Packaged foods commercially sterilized may be stored in hermetically sealed containers at room temperature for up to 2 years (Featherstone, 2015).

**FIGURE 2:**
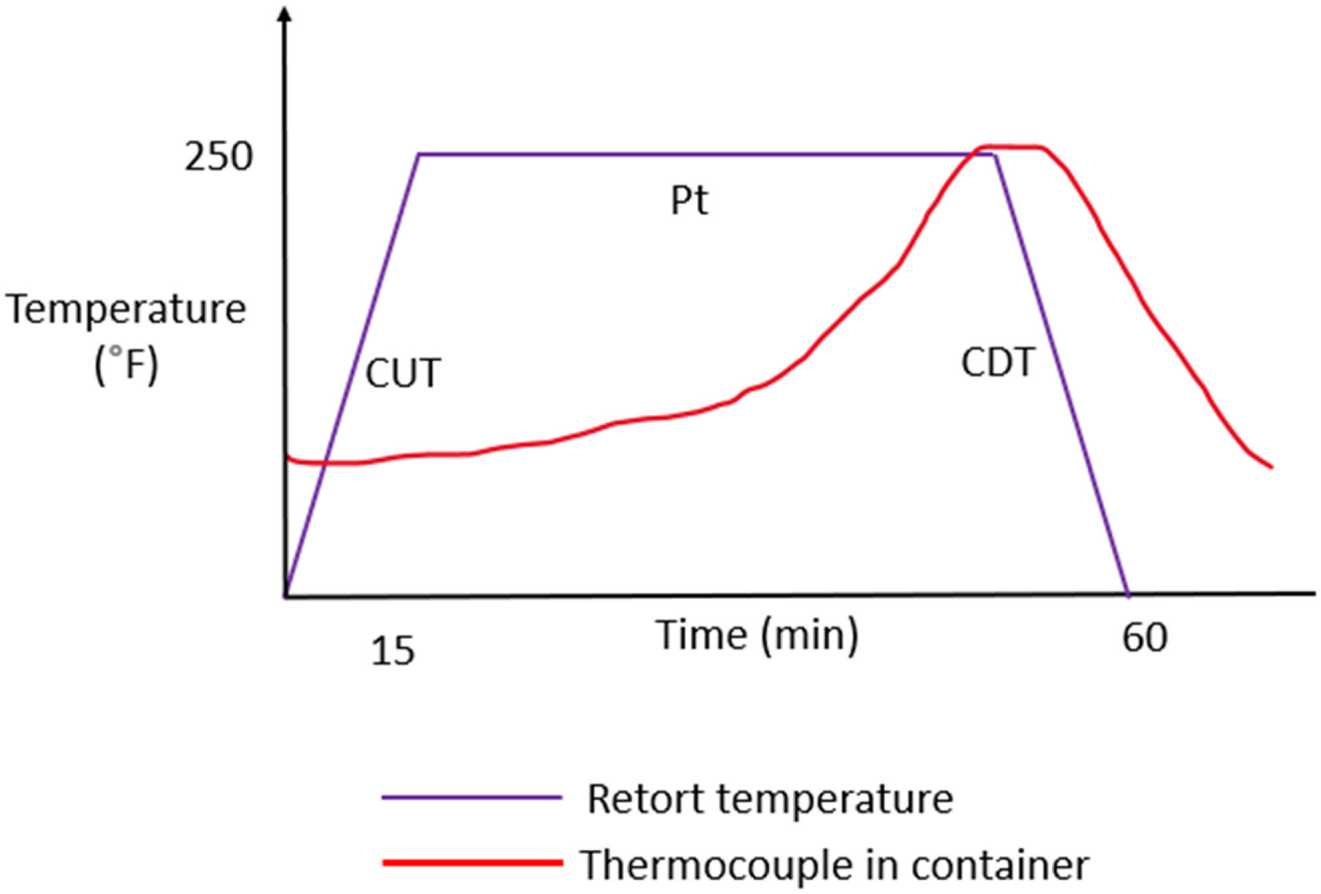
Basic temperature curve for retort thermal processing and profiles. CDT, come‐down time; CUT, come‐up time.

The sterilization procedure involves a three‐stage cycle (Figure 2). The first stage, CUT (come‐up time), is the time required for a high‐flow heating medium to reach the retort temperature of 240–250°F (approximately 115–121°C) and pressure required 15–20 psi (approximately 1–1.4 bar) above atmospheric pressure. In the second stage, *P*_t_ (holding or cooking stage), the retort maintains temperature and pressure to guarantee lethality; this varies depending on the target microorganism or anticipated microbial contamination. In the third phase, CDT (come‐down time), cooling water is added for a continued temperature decline. Excessive heat processing of food can be avoided by cooling it, which also prevents the development of thermophilic microorganisms. However, the cooling process can cause retort pouches to burst. This can be avoided by applying overpressure air during the cooling procedure, which helps to maintain package integrity and avoid container deformation (Mosna & Vignali, 2015).

## 3 RATES OF RETORT PROCESSING

The classification of retorts can be either batch or continuous (as shown in Flow Chart 1). The selection of the type of retort to be used depends on various factors, including the type and size of containers being processed and the quantity of the product. In general, small‐ to medium‐sized facilities may need help to justify the cost of a continuous retort, while large canning factories with higher production volumes may find it more cost‐effective.

**FLOW CHART 1:**
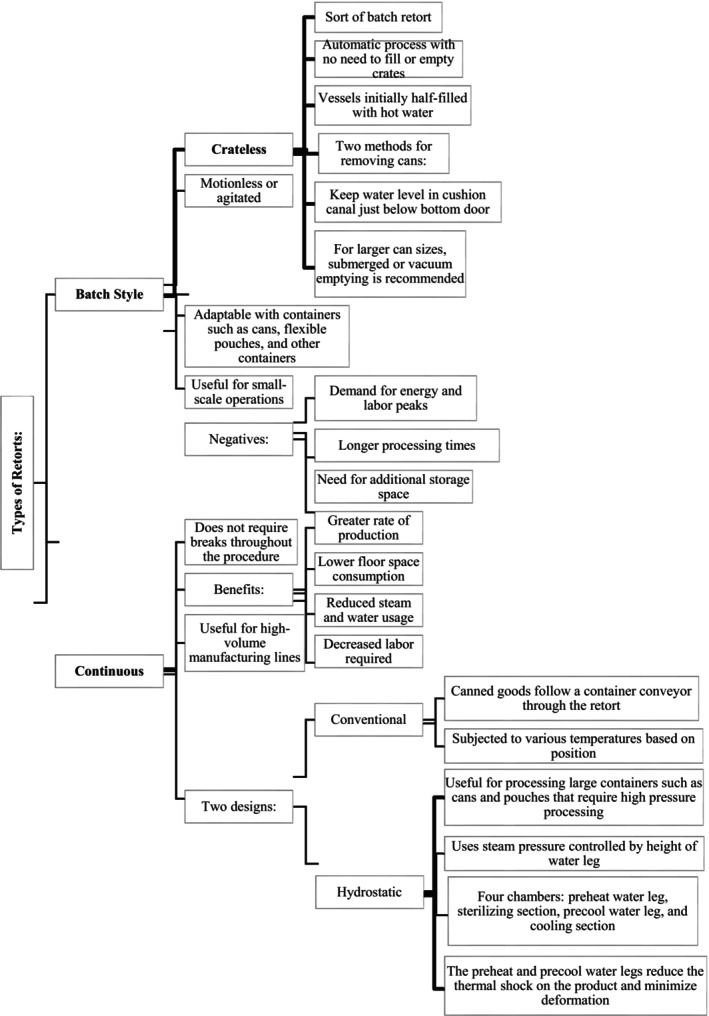
Types of retorts (Berk, 2018a; Holland, 2008).

### 3.1 Batch

**FIGURE 3:**
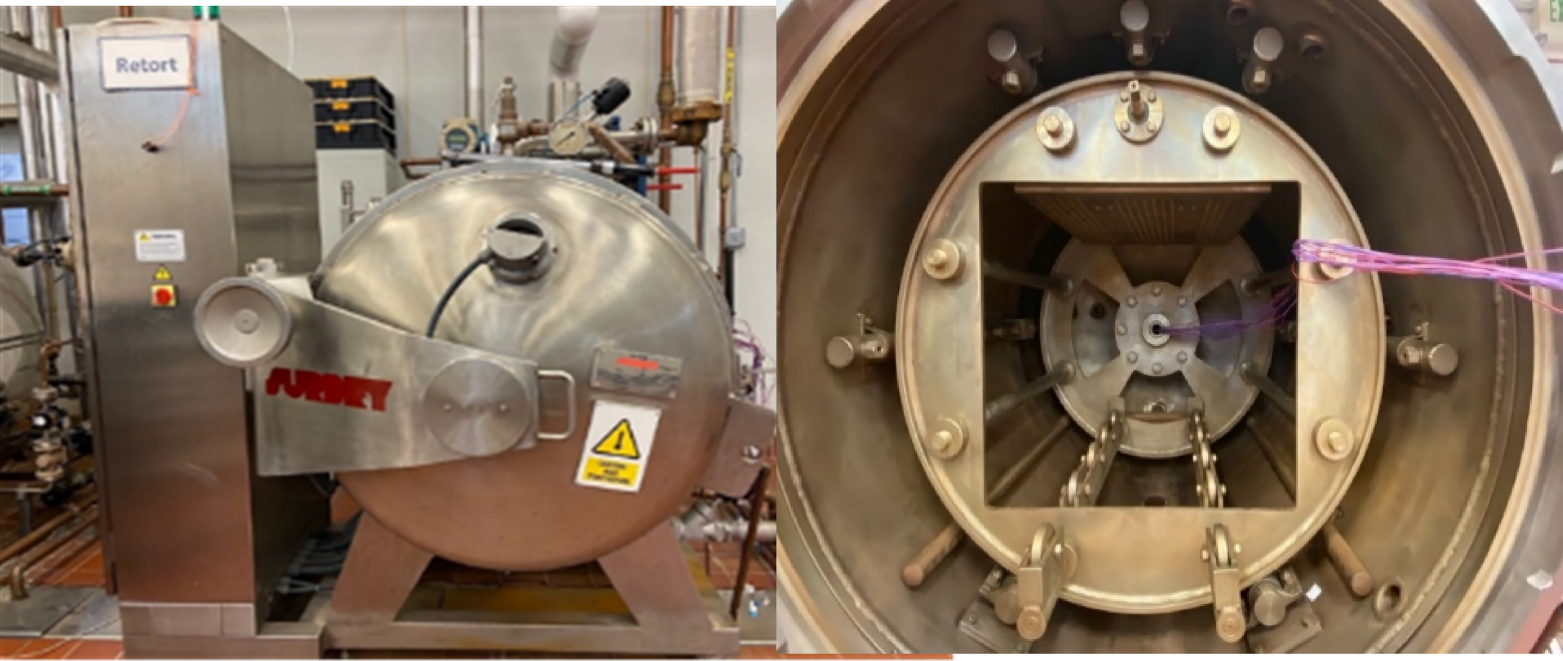
Retort Surdry Model APR‐95 (Photo courtesy of Cryovac® Flavor Mark® Retort Laboratory, Clemson University, SC).

The conventional method for sterilizing canned food in steam batch retorts over the past 75 years has been through a batch process (Alonso et al., 1993). The product is placed into the retort and thermally treated with a time gap between processing runs. Batch retorts can be motionless or agitated and can be horizontal or vertical (Figure 3).

**FIGURE 4:**
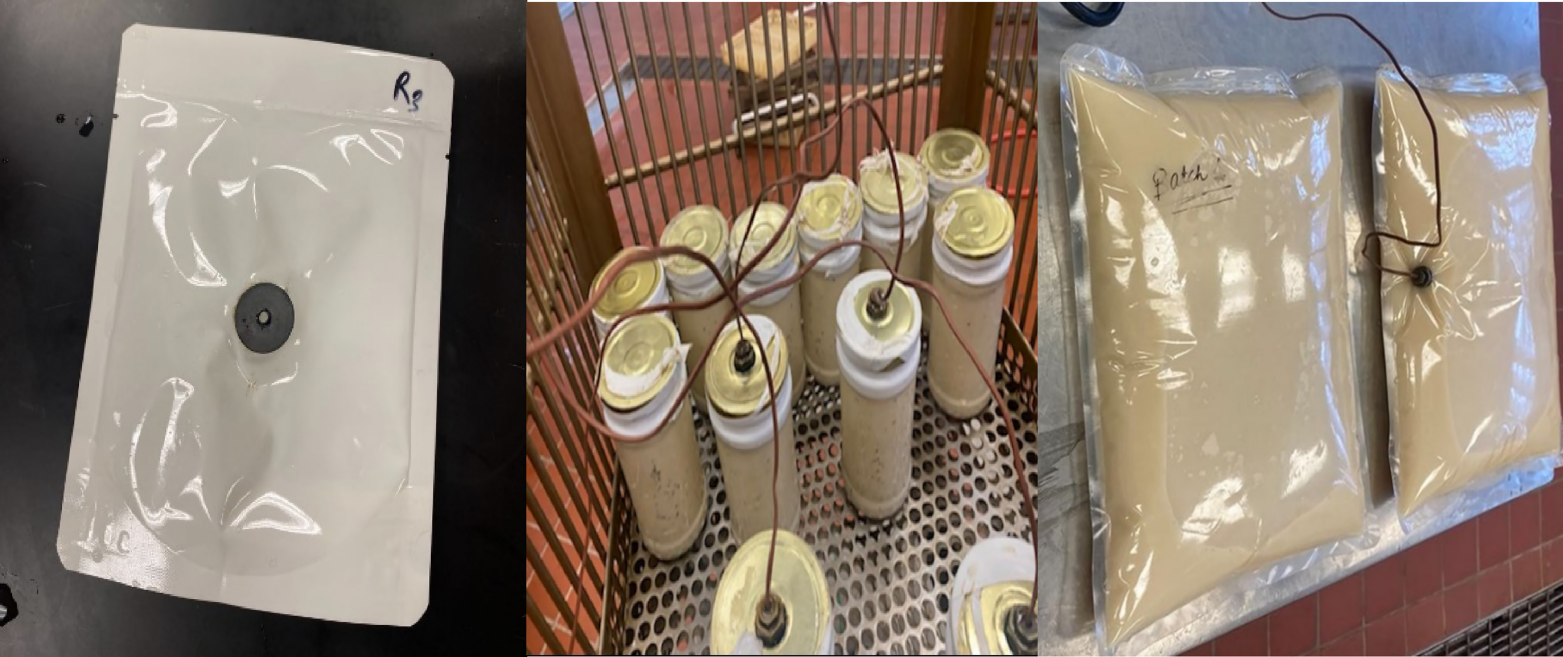
Retort food samples (Photo courtesy of Cryovac® Flavor Mark® Retort Laboratory, Clemson University, SC).

They generally come equipped with a pressure gauge, a thermometer, and automatic control systems. Batch retorts are adaptable with containers such as cans, flexible pouches, and other containers with only minor changes to the processing conditions (Figure 4). However, batch retorts have some limitations, such as peak energy and labor demand, underutilization of plant capacity, and underutilization of individual retorts (Peesel et al., 2016).

The venting step consumes the most energy in the first few minutes of the processing cycle. Large‐scale manufacturers that utilize retorts may have issues with energy consumption because retorts must run on a staggered timetable to minimize peak energy demand. To optimize energy usage and processing in batch procedures, researchers have investigated utilizing time‐variable retorting temperature processing (TVRT). According to studies, nutrient retention at a constant retort temperature is comparable to that of a TVRT process (Simpson et al., 2006). However, the processing time is significantly reduced with the use of a TVRT process.

The crates used in batch processing can also impact the process. They hold the various retorted goods and allow for faster filling and removal (Teixeira, 2019). Separators between the container layers must minimize resistance to flow because heat is transferred via a combination of forced water circulation and container mechanical movement (Featherstone, 2015). Separators are utilized to create separation between container layers, limit the expansion of semi‐rigid and flexible containers, and provide support and circulation channels in thin containers (Llosa Sanz, 2017).

Crateless retorting is a batch retort in which time is not taken to fill or empty crates as compared to regular batch operation, and the process is managed automatically. The vessels are initially half‐filled with hot water, and the cans are loaded from the top. When the cans are loaded, the water in the vessels acts as a cushion for them. There is a pause between thermal processing cans (Kou et al., 2019; Teixeira, 2019). The retort is filled with steam and pushed out through a drainpipe due to the air in the equipment expanding. After that, the device functions similarly to conventional steam–air processing. There are two methods for removing cans from a retort of this type. In the first technique, the water level in the cushion canal must be kept just below the bottom door of the retort. For larger can sizes, submerged or vacuum emptying is recommended. When utilizing this method, remember that the cushion canal's water level must be maintained above the retort's bottom door (Berk, 2018b).

### 3.2 Continuous

**FIGURE 5:**
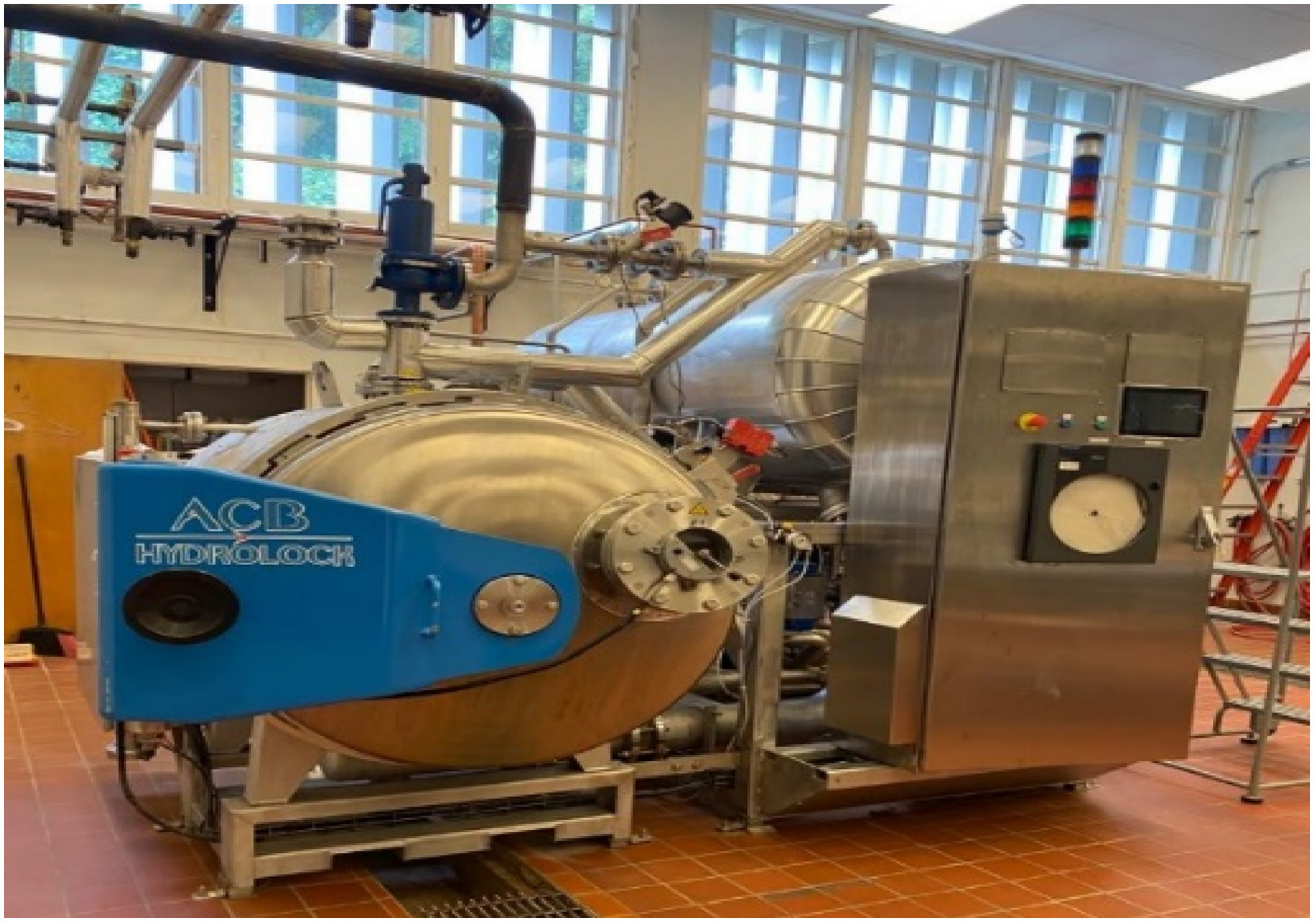
Retort ACB Hydrolock (Photo courtesy of Cryovac® Flavor Mark® Retort Laboratory, Clemson University, SC).

The process of continuous retorting does not require breaks and offers several advantages over batch processing, including cost savings, reduced labor, energy consumption, and equipment downtime. This is particularly beneficial for high‐volume production lines that do not require frequent adjustments to processing conditions or container sizes. In the conventional method, canned products move on a container conveyor through a retort, where they are exposed to different temperatures based on their position. Hydrostatic retort is another design used for continuous retort processing. This method involves using steam pressure that is controlled by the height of the water leg. These retorts can be of various sizes, and sometimes, the legs can be held outside the retorting facility. The hydrostatic retort typically includes four chambers: a preheat water leg, a sterilizing section, a precool water leg, and a cooling section (Figure 5). Hydrostatic retorts can operate at different temperatures and pressures, making them versatile thermal processors (Chen et al., 2008).

## 4 METHODS OF HEATING AND AGITATIONS

The three primary retort processes are steam, falling water, and full water immersion. Each of these categories has subcategories, such as steam–air, steam spray, water spray, and half‐immersion (Holland, 2008). In each case, a form of water is used, sometimes in combination with air, to transfer thermal energy to the product (as shown in Table 1). Pressure is applied in all retort methods to increase the boiling point of water and allow for higher temperatures. Some methods, such as those using air, also create an overpressure to prevent container deformation (Mosna & Vignali, 2015).

**TABLE 1:** Methods of heating.

Method	Process	Advantages	Disadvantages
Water Immersion	Whole or partial immersion of container in heated water through processing	Suitable for most products, water recirculation ensures uniform heat distribution, can control container float, and immersion is more efficient at high speeds	Requires water circulation to ensure uniform heat distribution, increased basket manufacturing cost for complete immersion, less adaptable
Steam–air	The method uses a mixture of steam and air	Offers rapid heating, which results in shorter process times compared to traditional steam retorts	Independent control of temperature and pressure in the process requires a high degree of technical knowledge
Saturated steam	Injection of saturated steam, pure steam	It requires careful control to ensure it does not overheat or underheat the product	Requires evacuation of air
Water cascade/spray	Water sprayed under pressure on top trays of retort to create indirect steam heating	Evenly distributed indirect steam heat, popular in industry, similar to water spray	Requires pressurized water supply

*Source*: Holland (2008) and Mosna and Vignali (2015).

The type of heating used can affect the rate of heat transfer and processing time. For example, Zhu et al. (2022) found that heating medium had no significant effect on processing time but did impact on heat transfer rate. Additionally, Ramaswamy and Grabowski (1999) reported that the type of heating medium used significantly affected the heating rate index in samples of Pacific salmon. These findings suggest that selecting the type of heating medium is crucial for improving processing efficiency and reducing production costs while maintaining high‐quality canned food.

### 4.1 Methods of heating

#### 4.1.1 Saturated steam

A saturated steam retort is a simple type of autoclave that is usually vertical and uses steam as a heating medium. It is crucial to remove air during the ventilation stage using venting, an injected steam method, to prevent cold zones from forming. The use of saturated steam in retort processing has been found to be cost‐effective and energy‐efficient compared to other heating methods. However, this type of retort poses several challenges to processors, such as pressure fluctuations, which make it challenging to process pouches, semi‐rigid containers, and trays without package distortion or cold spot formation.

#### 4.1.2 Water cascade/spray

The cascading water technique is a type of indirect steam heating where water is sprayed under pressure onto the top trays of retort carts. This method evenly distributes heat across a large volume of water, allowing heat to pass through the sidewalls of the container as water passes through the containers. Studies have shown that concerns about insufficient temperature dispersion are unfounded, as the variation in processing lethality throughout the retort is minor, and there are significant temperature variations during the cooking period. Furthermore, atomizing nozzles positioned around the chamber can be used to enhance the retorting process by providing excellent heat transmission and quick heating without requiring a fan to circulate air.

#### 4.1.3 Water immersion

Water immersion is a commonly used retort process (Adepoju et al., 2017), where water is first heated and then pumped into the retort for processing. The container is typically fully submerged in water during processing, with overpressure created by blowing air or steam for improved heat transfer patterns. However, in certain situations, such as with half‐immersion, packages are only partially submerged in water (less than half). This can be advantageous for high rotational speeds, as the cage creates less turbulence (Featherstone, 2015; Holland, 2008).

Water is recirculated during the heating process to ensure uniform heat distribution throughout the retort. Researchers have found that the position of products in a tray and the tray's height can impact heat transfer coefficients (Ramaswamy et al., 1991). Poor circulation can result in insufficient heat transfer.

Controlling the float of packs can be a challenge, and pouches and trays have often impeded this process, increasing basket manufacturing expenses and reducing adaptability. Half‐immersion occurs when the vessel is half‐filled with water, and part of the rotation occurs in and out of the water. This method is beneficial for high rotational speeds because the cage creates less turbulence. Manufacturers such as Stock Inc., FMC, Lagarde, and Lubeck produce this system (Holland, 2008).

#### 4.1.4 Steam–air

The use of steam and air is another popular medium for thermal processing. Lagarde Autoclaves patented this process in 1972, and it is highly efficient. The process differs significantly from the steam retort, with a horizontal vessel that has quick‐opening doors for easy loading and unloading of baskets, forced steam circulation, and, most crucially, independent control of temperature and pressure (Holland, 2008). Steam and air are continuously supplied to the retort vessel to create a homogeneous mixture. When water and steam are combined, the retort is pressurized, resulting in an overpressure situation that causes continual venting. This continuous flow of heated steam past the containers prevents the formation of cold spots (Adepoju et al., 2017). The technique was initially designed for flexible and semi‐rigid containers, such as military rations in aluminum foil packs, but it has since been adopted for pouches and ready‐to‐eat food (Holland, 2008).

Early studies of steam–air processing media revealed the possibility of producing a non‐homogeneous mixture of steam (Ramaswamy et al., 1991). Europe and Japan used the method for commercialization for a long time before North America adopted it. Later research revealed that the heat transfer pattern would be adequate as long as there was enough mixing (Ramaswamy et al., 1991).

The type of airflow is determined by the retort's design. Positive‐flow retorts create upward flow and are intended for vertical retorts. Horizontal flows are used in Lagarde retorts, which are intended for horizontal retorts. A study comparing these two styles found that the overall mean heating rate index for positive flow was only slightly higher than that of the Lagarde retort (Ramaswamy & Tung, 1988).

#### 4.1.5 Steam water spray

In 1983, Surdry of Spain patented the atomizing steam and water method, which is a relatively new batch‐retorting technique. This method utilizes atomized air to provide excellent heat transfer to rigid containers, and a fan is not used to circulate the air. Instead, water is drawn from a pump and combined with condensate from the retort's center and recirculated condensate before being supplied directly into the chamber via atomizing nozzles located around it. While the atomizing nozzles allow for rapid heating, they tend to obstruct water flow during cooling, resulting in longer processing times compared to cascading water, immersion, or water spray retorts (Holland, 2008).

### 4.2 Methods of agitation

The first type of retort used for canning, known as static or still retorts, is commonly used for liquid food products and does not produce any agitation in the containers. However, this approach has some limitations, such as slow heat penetration creating different temperature zones within the package, which can lead to overcooking, uneven texture, and flavor.

An alternative to overcome this issue is through agitation. Agitation can help decrease heat damage and speed up heat penetration, leading to improved heat transfer and fewer cold spots, which ultimately results in more efficient thermal processing. Different agitation techniques (as shown in Flow Chart 2) include end‐over‐end, fixed axial, biaxial, and reciprocal agitation. When the container is agitated, air bubbles move around it, leading to a more uniform distribution of heat.

**FLOW CHART 2:**
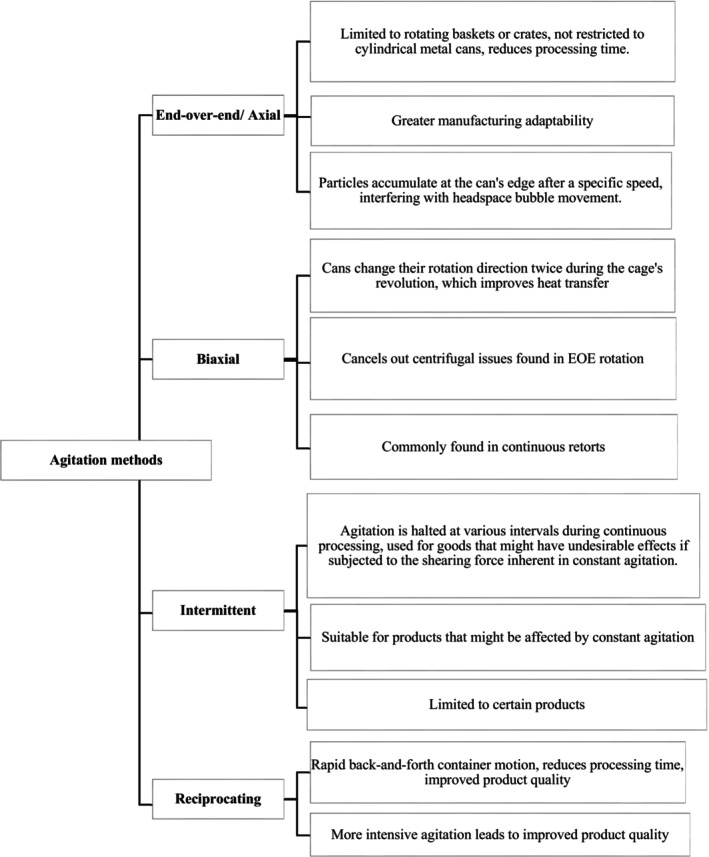
Agitation methods.

#### 4.2.1 End‐over‐end/axial

End‐over‐end (EOE) or axial mode pack rotation is a popular technique that has gained popularity in recent years, where packages are rotated in limited‐rotation baskets or crates. This batch method allows for greater manufacturing adaptability and is not restricted to cylindrical metal cans (Zhu et al., 2022). Commercial rotary retorts such as Sterilmatic, Steristar, and Rotomat operate on EOE rotation. However, this technique has several drawbacks. Researchers have discovered that particles accumulate at the edge of the container after a specific speed, which interferes with the headspace bubble movement within the can due to the equal centrifugal and gravitational forces within the can. The EOE rotation technique involves rotating cans in a circular motion, which requires headspace in the container (Singh & Ramaswamy, 2015).

#### 4.2.2 Biaxial

Another method for agitation is biaxial agitation, which is commonly used in continuous retorts. In this method, metal cans change their direction of rotation twice during the revolution of the cage, which cancels out the centrifugal issues found in EOE rotation and improves heat transfer (Dwivedi & Ramaswamy, 2010; Singh et al., 2015). The use of horizontal axial rotation for agitation dates back to the 1920s, when cans were rolled to produce agitation. In the 1950s, vertical rotation was suggested as a means to improve heat transfer in canned products.

#### 4.2.3 Intermittent

Intermittent agitation involves intermittently stopping the agitation during continuous processing. This technique is used commercially in the FMC Sterilmatic, which processes cans using intermittent axial rotation (Tattiyakul et al., 2002). This method is appropriate for products that may be harmed by the shearing force inherent in constant agitation. When starch granules are heated, for example, they expand and form a thickened dispersion. The dissolution of granule components in the matrix could be caused by the heating and shearing of starch granules in the matrix (Tattiyakul et al., 2002). This results in hydrogen bond breakdown, depolymerization, and decreased viscosity.

#### 4.2.4 Reciprocating

Reciprocation agitation, which involves rapid back‐and‐forth motion of containers, is a promising type of agitation. Walden invented the first reciprocating steam cooker in 1999, but Gerber invented it in 1938. Reciprocal agitation has grown in popularity since the invention of the Shaka and Gentle Motion retorts. The optimal shaking rate for a reciprocal agitation sterilization system is determined by factors such as the food product, container size and shape, and the desired level of microbial inactivation.

The Shaka process is used only for liquid products with fast agitation rates greater than 1 Hz, whereas the Gentle Motion method is used for liquid‐particulate meals with slower agitation rates. Many recent studies on this technique have been conducted, including investigations into reciprocation intensity, amplitude, frequency, container placement, headspace, and particle size. According to one study, the most significant effect on thermal transfer was reciprocation speed, followed by amplitude and frequency. Another study discovered that thermal processing canned shrimp with reciprocal agitation resulted in superior product quality, improved process parameters, and potential energy savings. At all agitation speeds, reciprocal agitation technology resulted in a shorter process time than static retorts for achieving the target F0 value and improved product quality. This is due to the combination of gravity and horizontal acceleration in the processing (Walden & Emanuel, 2010).

#### 4.2.5 Oscillating

According to a study conducted by McNaughton in 2018, the use of oscillating motion during thermal processing can significantly reduce the average calculated process time by 10%–27% compared to static mode with the same thermal process parameters. The study utilized a two‐basket water spray retort and calculated process times using Ball's formula method. The oscillating method utilized a speed of 10.5 RPM with an oscillating angle of 15°. The study also observed that depending on the amount of residual air in the pouch, there was a significant difference in the average slope value of the heating curves within the static motion, within the oscillating motion, and between the static and oscillating motions (Flow Chart 2) (Holland, 2008; Singh & Ramaswamy, 2015).

## 5 THERMAL MONITORING AND DATA ANALYSIS IN HEAT DISTRIBUTION AND PENETRATION

### 5.1 Monitoring and data collection

**FIGURE 6:**
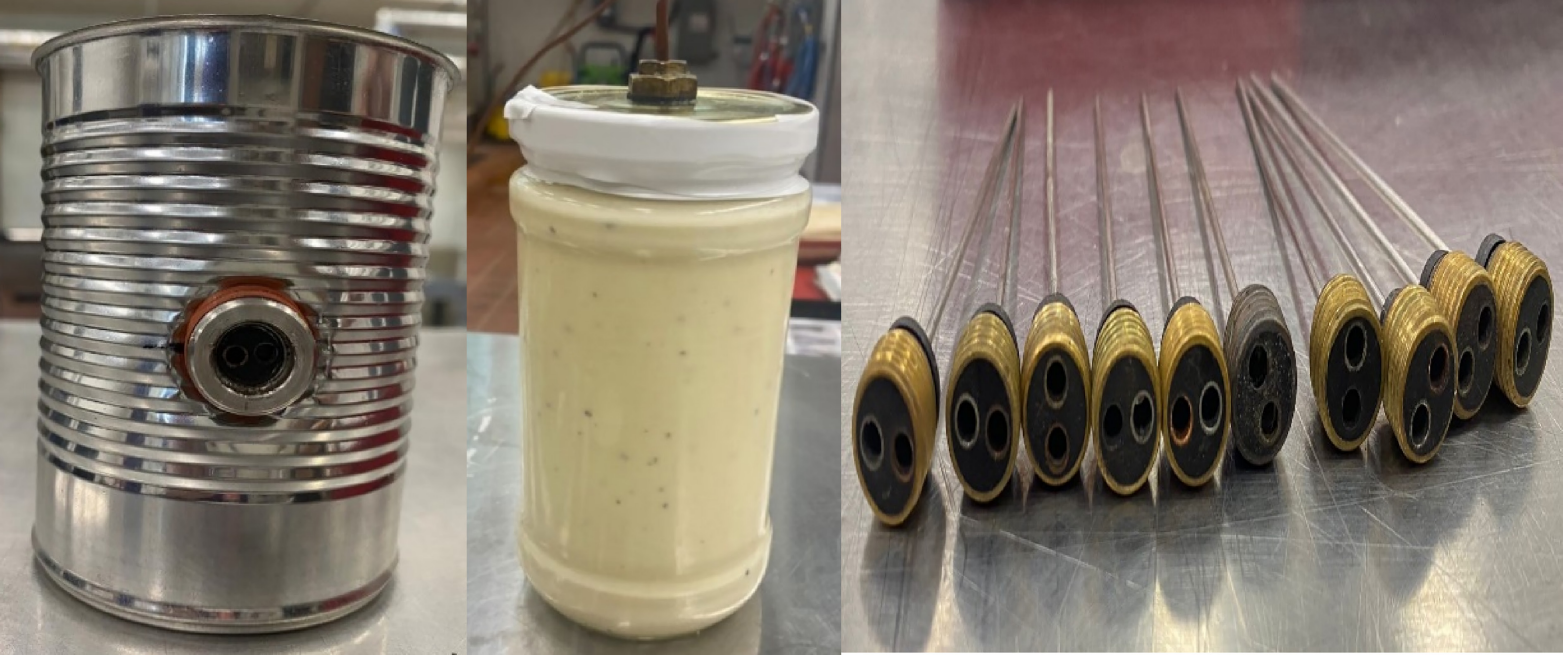
Assemble thermocouples in glass jars and metal cans (Photo courtesy of Cryovac® Flavor Mark® Retort Laboratory, Clemson University, SC).

Temperature monitoring systems use various sensors throughout the retort to collect data for temperature distribution and heat penetration studies. Thermocouples (TMD) are the most widely used instruments made from two dissimilar metals connected at two junctions. The T‐type (copper constantan) and K‐type (chromel constantan) are the most popular types (Forney & Fralick, 1994). They are widely accepted due to their low cost, precision within the desired temperature range, responsiveness, and ability to be assembled on different types of containers, such as jars and pouches, as shown in Figure 6 (Berrie, 2001).

Modern data loggers are equipped with sensors to collect data and can be either connected or wireless. They are multi‐channel systems with digital responses, allowing readings to be sent directly to a laptop for collection and storage (Awuah, Khurana, et al., 2007; Berrie, 2001).

Thermal processing is typically carried out with the help of an onboard control system or a computer. The LOG‐TEC Process Management System was the first commercial system introduced, and it is still in use today. The HP‐85 desktop computer with an HP‐3497 datalogger was the first computer used for this purpose (Gill et al., 1989). Regulatory agencies such as the FDA/USDA in the United States and the FSA in the United Kingdom recommend additional data collection to improve thermal processing control and protect customers. A host computer with customized product recipes is used to store data and meet the regulators' requirements for data sent electronically in a specific format and by a PC. This document can then be filed, either directly from the computer or via a remote PC, in an easily readable form that requires no extra software (Mosna & Vignali, 2015).

The IFTPS (2014) states using thermocouples, wireless data loggers, and other comparable devices to monitor temperatures during thermal processing. All instruments used must be of high precision and size, as well as be available in sufficient numbers, to ensure proper and safe observation of the process environment. Before conducting experiments, all TMDs (temperature‐measuring devices) should be carefully calibrated and tested under the same retort conditions desired for the process (IFTPS, 2014; Llosa Sanz, 2017).

### 5.2 Distribution and penetration of heat

**FIGURE 7:**
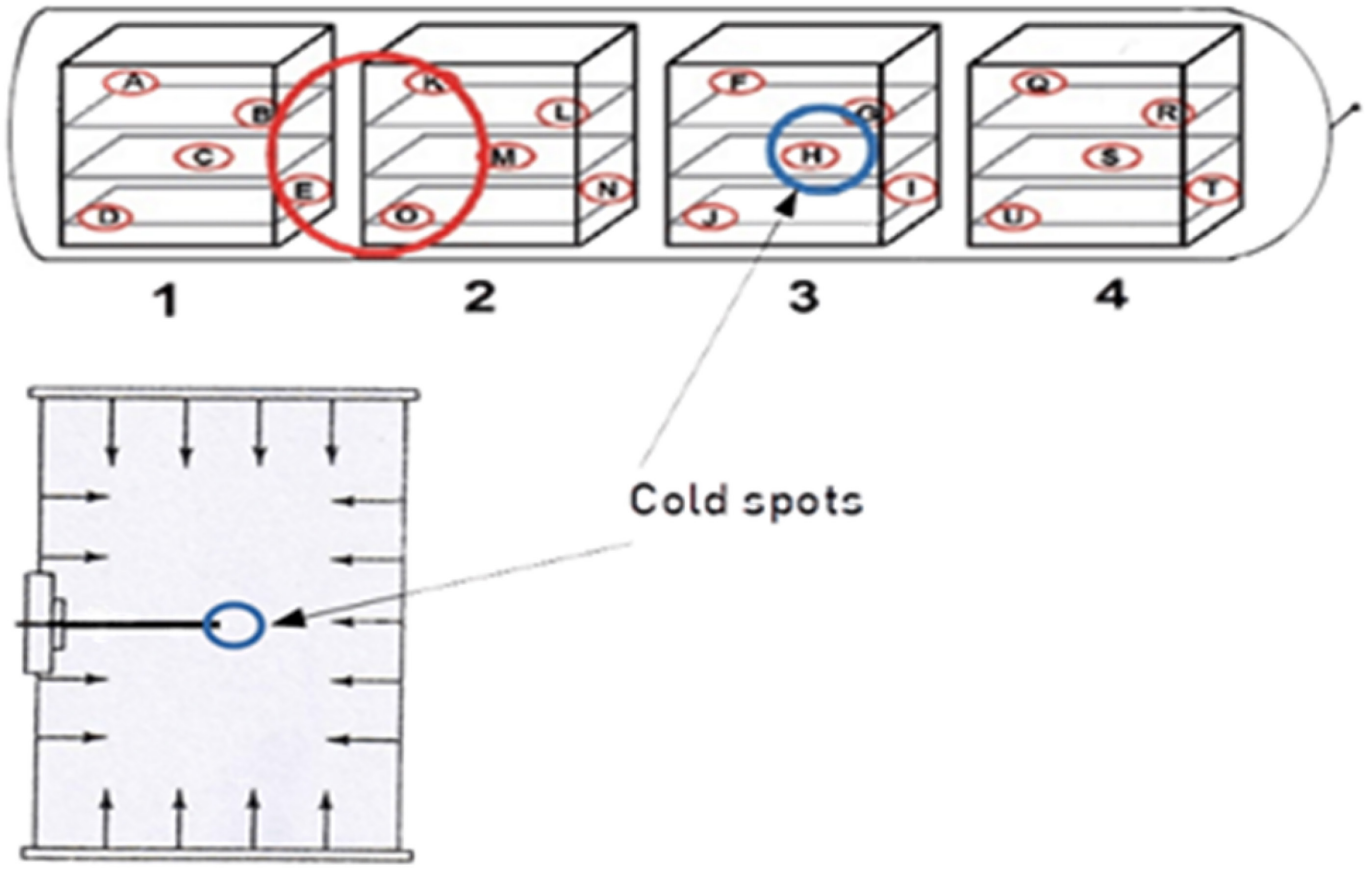
Non‐uniformity during heat distribution and penetration (Axitherm, 2023).

Thermal non‐uniformity is a concern that can result in cold spots (Figure 7), which lower the microbiological safety of the product. To address this, heat distribution and heat penetration are two important variables that need to be considered during thermal processing. Heat distribution refers to the delivery of heat by retort equipment to the product area. In contrast, heat penetration refers to the alteration in the supply of heat from the product area to its coldest point (areas within the product receive less heat relative to its adjacent regions).

**FIGURE 8:**
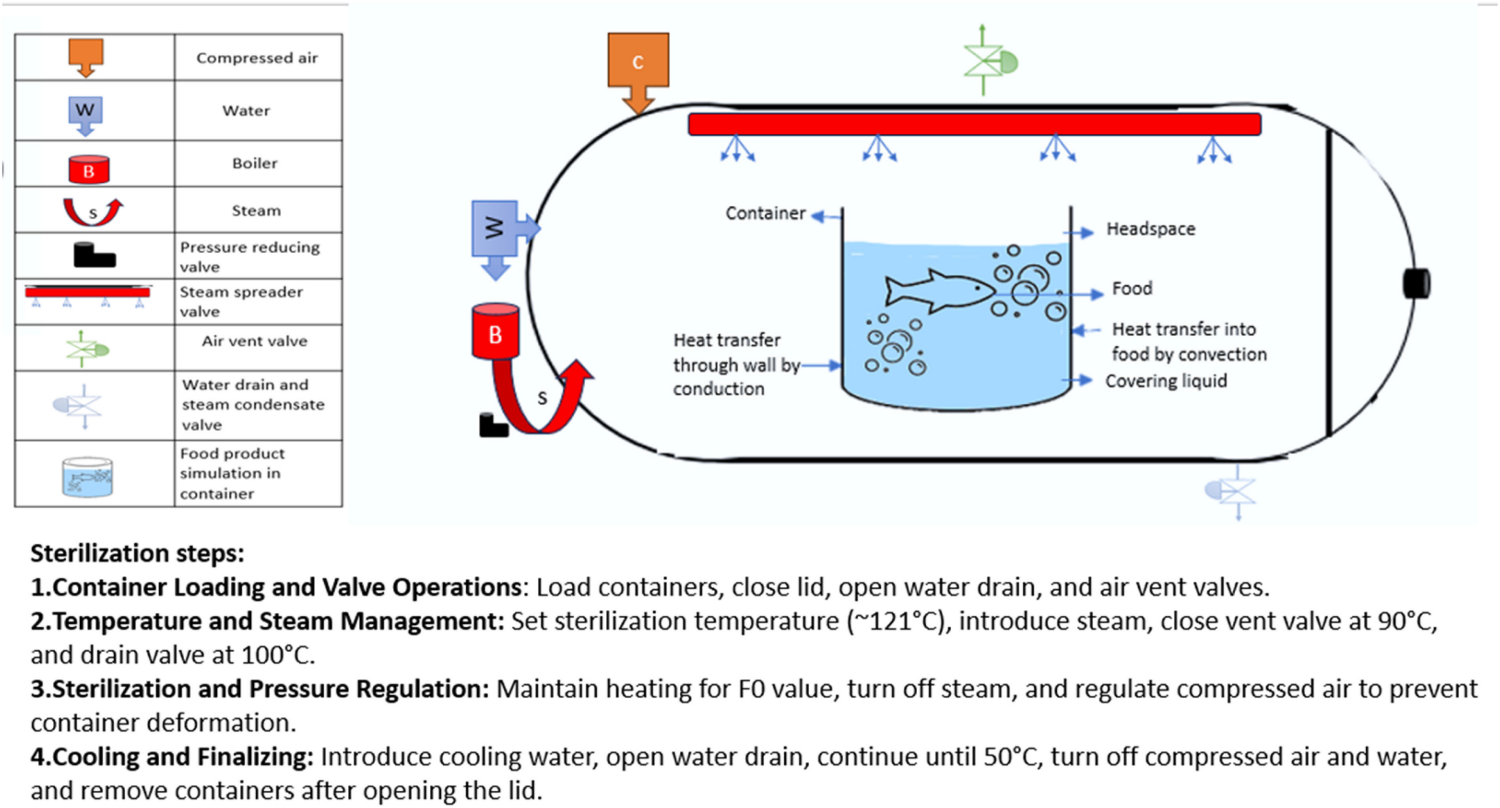
Schematic of a sterilization in retort process.

Designing the sterilization process requires information on the time–temperature distribution profile within the mass of the food product, as well as the kinetics of thermal inactivation, such as thermal death and thermal destruction. However, the difficulty of heat transfer phenomena during retorting is due to the fact that foods can be a mixture of solid and agitated liquids (Figure 8). Additionally, certain types of foods, such as starch or protein‐based foods, can significantly change their rheological characteristics during processing (Zhu et al., 2022).

## 6 PROCESS CALCULATIONS TO ENSURE FOOD SAFETY

According to Powers et al. (1962), public health concerns are caused by “improper application of the technique” rather than the procedure itself. Lúquez et al. (2021) reported that from 1950 to 2005, 34 instances of foodborne botulism associated with commercially processed foods were reported by the Centers for Disease Control and Prevention (CDC), and only four of these occurrences resulted from poor canning techniques. However, in 2007, ten cases of botulism were linked to commercially marketed canned hot dog chili, indicating inadequate safety measures at manufacturing plants that allow botulism spores to remain under harmful conditions. Home‐canned foods are more commonly associated with botulism than commercially canned goods, which are usually prepared following proper procedures (Juliao et al., 2013). Despite the implementation of retort technology, there have been significant safety concerns due to bacterial and spore contamination (Gill et al., 1989).

Thermal process calculations are crucial to ensuring the proper process and safety of food products. Bigelow's general approach, which he developed in the early twentieth century, and Ball's semi‐analytical approach are commonly used to calculate the lethality value of thermal processes. The effectiveness of thermal processing depends on several factors, such as processing temperature, environmental conditions, microbial properties, and product characteristics.

The F0 value, introduced by Ball (1927), is essential in thermal processing, as it describes the log reduction of bacteria during the retort process at a predefined location. The heat transfer coefficient (*h*) and overall heat transfer coefficient (*U*) are also critical parameters to consider during thermal processing. The thermal death rate of bacteria follows a first‐order semilogarithmic rate, which means that a product cannot be entirely sterilized but can be commercially sterile. The mathematical values used to express thermal resistance during processing, such as the decimal reduction era (*D*‐value) and the (*Z*‐value), are uniform across different types of thermal processing.

Dr. Robert Bigelow created the first method to calculate the basis of minimal safe sterilization, known as Bigelow's general approach (Bigelow & Esty, 1920), which has been widely used in practice since then (Awuah, Ramaswamy, & Economides, 2007; Simpson et al., 2003). This technique relies on real‐time monitoring of the coldest point in thermal processes using computer programs to obtain the lethality value of a process. Although this method was found to be extremely time‐consuming, difficult, and unworkable due to the lack of programmable calculators or personal computers at the time (Simpson et al., 2003), it is still useful today.

In response to the need for a more efficient method, in 1923, scientist Ball (General method) unveiled a semi‐analytical approach for thermal process calculation to the scientific community. This alternative technique employs theoretical models that predict thermal survival and heat penetration equations based on physical theories (Simpson et al., 2003).

The determination of the heat transfer coefficient (*h*) and overall heat transfer coefficient (*U*) are crucial parameters in heat transfer analysis. Empirical equations that use dimensionless quantities are often used to calculate these values (Nelluri et al., 2022). Broken‐line heat penetration curves, which resemble straight‐line heating curves, are characterized by a heating lag factor (jh), two heating rate factors (fh1 and fh2 for the first and second linear segments), and a breakpoint time (xbh) (Bigelow & Esty, 1920; Zhu et al., 2022). These parameters can be estimated through a graphical analysis of a heat penetration curve or a computer program developed by (Denys et al., 1996).

### 6.1 Bacterial inactivation

One of the primary aims of thermal processing is to inactivate bacteria. To produce safer and more shelf‐stable products. Retorting has been demonstrated to be an effective and cost‐efficient technique for producing food (Verheyen et al., 2021). However, the excessive microbiological safety margins of the thermal processes employed often lead to nutrient loss and sensory dulling due to higher temperatures and times. The rate of thermal death of bacteria is a time/temperature process with first‐order semilogarithmic rates. This implies that a sterile product cannot be manufactured, but commercially sterile products can. The mathematical value definitions are the same for each type of thermal processing. Decimal reduction time (*D*‐value) and the *Z*‐value express thermal resistance in processing. The *D*‐value is the duration of heat treatment at a specific temperature necessary to kill 90% of the microbial population. The *Z*‐value is the temperature change needed to shift the *D*‐value by 1 log unit (Ates et al., 2016; Zhu et al., 2022). The efficacy of thermal treatment is determined by the processing temperature, pressure, target microorganisms, and product characteristics (Tavman et al., 2019).

The Fo value, established by Ball (1927), is one of the most crucial values in thermal processing. It describes the log reduction of bacteria that would occur during the retort process at a specific spot (Awuah, Ramaswamy, & Economides, 2007). Stumbo established the D121.1 value of 0.21 min at 121.1°C (250°F) as equivalent to 2.52 min, establishing a Fo value of 2.52, based on the acceptable probability of survival is not more than 1 in 10^12^ containers. Since 1965, a minimum “botulinum cook” Fo value of 3 min has been determined and is still used today for low‐acid canned items (Bean et al., 2012).

Testing and validation of new thermal processing technologies require time and replication. As a result, many researchers have utilized food substitutes rather than real food samples to evaluate their procedures (Verheyen et al., 2021). However, the current outbreak demonstrates that this method must be analyzed. In other words, there is a lack of experimentally confirmed microbial inactivation with actual food matrixes via reciprocal agitation. Only Ates et al. (2014) have studied this agitation method using a fish soup model to analyze *Listeria innocua* inactivation, and the effectiveness of reciprocity agitation in microbial inactivation compared to a static retort process using sausage and chicken soup samples treated with *L. innocua* and then thermally processed. The shaking process provided equivalent lethality in a significantly shorter time than the static retorting technique. Other researchers investigated the thermal inactivation rates of *Listeria monocytogene*s under shaka agitation. This study also looked at the impact of viscosity and fat content (Verheyen et al., 2021). It was found that at the 20% fat emulsion, the fat created a protective environment for bacterial cells. This only led to local changes in heat transfer but did not affect the final *L. monocytogenes* reductions.

### 6.2 Spore inactivation

The presence of spores in a food product can pose a significant risk to consumers; therefore, their elimination is crucial. Spores are known to be resistant to heat, radiation, and various chemicals, making their elimination challenging (Ates et al., 2016). However, thermal processing can weaken spores, and some bacterial spores can be eliminated using high‐pressure conditions (>1000 MPa). Unfortunately, such high‐pressure conditions are not feasible in the food sector due to practical limitations (Ling et al., 2015).

Research has shown that spore inactivation can vary based on the retort processing technique employed. Different processing techniques, such as reciprocation, static, and high‐pressure–temperature treatments, have been investigated for their effectiveness in spore inactivation (Ates et al., 2016). The results indicate that spores of *B. subtilis* can be inactivated at significantly lower temperatures with reciprocation than with static processing. For instance, the agitation method was able to achieve a 7‐log reduction of spores after 17 min, whereas the static processing technique required 53 min to achieve the same result.

### 6.3 Critical factors

In retort processing, various parameters must be examined and controlled to ensure the safety and quality of the final product. These parameters include process variables such as the initial temperature of the product, formulation, variance in ingredient weights, packing density (which should be 5%–10% more than the nominal weight of the product), drained weight (maximum expected under production circumstances), and viscosity in semi‐liquid or liquid products. Container parameters such as dimensions, headspace (when using rotary retorts), vacuum, number of residual gases, and maximum thickness are also important to examine and control (IFTPS, 2014; Llosa Sanz, 2017). Other variables such as the rotary axis and time from filling to processing are also necessary to monitor and control (MacNaughton et al., 2018; May & Chappell, 2002; Meng & Ramaswamy, 2007).

Furan formation in products can be reduced by using variable retort temperature profiles (VRTPs) and retortable pouches. VRTPs enable more precise thermal processing condition control, reducing processing time by 20%–30% and improving surface quality by 5%–15%. Thermally processed foods' quality and safety can be improved by minimizing surface overcooking and using retortable pouches that allow for rapid heat penetration. Furan formation can be reduced further by reducing overprocessing, adjusting process parameters, and employing kinetic models. The VRTP is an effective tool for reducing processing time, increasing quality retention, and reducing furan formation in foods (Fardella et al., 2015).

The headspace fingerprint was investigated by Grauwet and Shpigelman (2018) as a potential multivariate intrinsic indicator to monitor temperature variation during thermal in‐pack processes. The authors used broccoli puree as a case study to demonstrate the potential of this approach to provide real‐time monitoring of temperature variation in packaged foods.

## 7 NUTRITIONAL VALUE/QUALITY OF RETORT PROCESSED FOODS

Numerous research has been conducted to investigate the effect of retort processing on the nutritional content of foods. Barreiro et al. (1997) examined the kinetics of color change in thermally treated double‐concentrated tomato paste. Their findings suggested that heat processing significantly impacted the lycopene concentration, an essential nutrient in tomatoes. Moreover, they demonstrated that the lycopene degradation or transformation rate can be effectively quantified using sophisticated kinetic models. This study underscores the criticality of temperature regulation in preserving both tomato‐based products' visual appeal and nutrient content. Further research by Singh and Ramaswamy (2015) explored the effects of reciprocating agitation thermal processing on the quality of green beans. Their study revealed significant alterations in the firmness and structure of the green beans, directly impacting their textural quality and potentially affecting nutrient bioavailability.

A related study by Reddy and Love (1999) provided a comprehensive review of the effects of thermal processing, including retort methods, on the nutritional value of vitamins and minerals in various food products. Their findings indicated that while specific vitamins degrade under heat, others may become more bioavailable, suggesting a complex interaction between nutrient types and processing conditions. Recent research by Lee and Shin (2023) delved into the physicochemical and sensory properties of retort chicken curry mousse fortified with branched‐chain amino acids. This study addressed sensory attributes such as taste and texture and showcased that retort processing can enhance the nutritional profile, particularly protein content, tailored to specific dietary needs. Additionally, Gokhale and Lele (2014) focused on the retort processing of traditional Indian foods, highlighting how to preserve the unique flavors and nutritional components during intense thermal processing. Their research is instrumental in ensuring these culturally significant dishes retain both traditional appeal and nutritional benefits post‐processing. These studies emphasize the versatility of retort processing across various foods and conditions, emphasizing its extensive application in the food industry. The various practical applications of retort thermal processing, as detailed in Table 2, demonstrate its crucial role in extending shelf life with safety standards.

**TABLE 2:** Retort processing summary of applications.

Food product	Retort conditions	Findings	References
Canned Beef	Temperature: 121°C; time: 20 min	Aroma was significantly similar to 121°C‐heated canned beef than 100°C‐heated beef. Higher amounts of pyrazine, 2‐methylpyrazine, and diacetyl in 121°C‐heated beef than in 100°C‐heated beef	Migita et al. (2017)
Winged Bean Seeds	Temperature: 245°F; F0 values of 5, 7, 9; time: 7, 10, and 14 min	Different F0 values affected moisture, protein, and carbohydrate content but not ash and fat content. Higher carbohydrate content at F0 9	Setiawan et al. (2023)
Fresh Milkfish	121.1°C in oil medium‐steam–air; F0: 7.87, time: 35 min	Faster heat penetration in steam–air application than in water immersion. Different processing effects in a dry pack and oil medium	Adepoju et al. (2017)
Chicken in Green Curry	Time: Varied (5–12 min); F0 = 4, temperature: 120°C	Process time was influenced by the heat capacity ratio of solid and liquid. Affected the texture and color of the chicken	Chysirichote et al. (2023)
Turkey Berries in Green Curry	Time: Varied (5–12 min); F0 = 4, temperature: 120°C	The process time was influenced by the heat capacity ratio of solid and liquid. No significant effect on turkey berries	Chysirichote et al. (2023)
Radish	Temperature: 120–150°C; time: 1–7 min	The highest heating rate among tested vegetables. L* values decreased as a function of increasing temperature and holding time, indicating reduced whiteness	Yu et al. (2023)
Carrot	Temperature: 120–150°C; time: 1–7 min	Hardness decreased significantly with increasing temperature and holding time. Reduced a* values indicating darkening	Yu et al. (2023)
Potato	Temperature: 120–150°C; time: 1–7 min	Hardness and browning increased with temperature and holding time	Yu et al. (2023)
Rohu Balls in Curry	Temperature: 121.1°C; F0 values of 6, 7, 9 min	Texture profiles such as hardness, springiness, gumminess, and chewiness decreased as the F0 value increased. The optimal F0 value for quality and safety was found to be 7 min. Decreased lightness (L* value) and increased redness (a* value) and yellowness (b* value) with increasing Fo values. The overall process time for Fo value of 7 min was 42.21 min	Majumdar et al. (2016)
RTE Rice (Plain and Chicken‐Flavored)	Time: 28 min, temperature: 121°C; F0 values of 6	Moisture content and lightness of rice products were maintained in both organic‐ and inorganic‐coated retort pouches during the 12‐week storage. No significant differences were found between the pouches for both plain and chicken‐flavored rice in terms of sensory characteristics, including aroma, color, glossiness, and stickiness. Acceptability remained high for both types of rice in both pouch types throughout the study period	Byun et al. (2010)
Shrimp (Louisiana Gulf Coast Brown Shrimp)	Agitation: 0, 45, 90, and 180 shakes per minute (SPM); F0 value of 6 min, temperature: 121.1°C; time: 30.0, 21.3, 19.0–17.3 min	Shelf‐stable shrimp were processed using high‐speed reciprocal agitation and compared with static retort processing. Total retort processing times decreased from 30 min at 0 SPM to 17.1 min at 180 SPM to achieve the same F0 value. Blanch yield was about 90%, and retort yield was 70%–75% after processing. Shear force texture increased with increasing agitation speeds, showing a range from 294 to 475 g‐F, with higher values indicating firmer texture. Surface sloughing and sedimentation increased at higher agitation speeds	Dixon et al. (2020)
Ribbon Fish in Oil and Curry	Oil pack: 47 min, Curry pack: 68 min; F0 value 12.84 (Oil), 10.8 (Curry), temperature: 115.6°C	Biochemical and sensory analysis showed slight increases in total volatile base nitrogen, tri‐methylamine nitrogen, thiobarbituric acid, and free fatty acid content after 5‐month storage, but within acceptable limits. Sensory quality remained good	Chandra et al. (2014)
Béchamel Sauce	Come‐up time: 2.0 min, Process F0 6: 3.5 min	Comparative sterilization times showing significantly shorter processing time for Béchamel sauce using the ShakaTM process compared to static and rotary methods	Walden (2008)
Buffalo Meat Nuggets	121°C, F0 12.13	Corn starch as a binder produced a more stable emulsion, higher shear force, and better sensory scores for texture and overall acceptability compared to other binders. Fat content was higher in products with corn starch	Devadason et al. (2010)
Fresh Green Peas	Varies with temperature (100, 110, and 120°C) and total processing time (27, 42, and 57 min)	Ascorbic acid retention was best at 100°C for 27 min with 85.40% retention, decreasing with higher temperatures and longer processing times	Garrote et al. (2009)
Pinto Saltillo Beans	Temperature: 121°C; time: 40 min	Lipid content decreased by 0.5% in two treatments and 0.3% in the third. Protein content was conserved, with phaseolin identified in all samples	Martinez‐Ceniceros et al. (2022)

## 8 RESEARCH COMPARISONS

### 8.1 Heat transfer (agitation)

When it comes to heat transfer, the speed and quality of the procedure may be affected by thermal transfer. Therefore, several studies have been conducted to determine the processing technique that enhances value. One study compared the heat input into corn starch during intermittent agitation to stationary and continual agitation values (Tattiyakul et al., 2002). The study found that the heat penetration rates during the first 600 s increased, and the temperature distribution was more uniform in the intermittent compared to the other methods.

Another study investigated how the heat transfer rates of EOE, free axial, and fixed axial rotation compared (Dwivedi & Ramaswamy, 2010). The study found that free axial rotation had greater heat transfer characteristics, followed by EOE and then fixed axial mode. Higher rotational speeds and retort temperatures resulted in higher U and lower heating rate index and lag factor for each agitation technique. A different study examined the lethality of canned potatoes during various forms of agitation (Rattan & Ramaswamy, 2014). In this experiment, free/biaxial, fixed axial, end‐over‐end, and static thermal processing were compared. The processing time was kept the same for each mode, and as a result, the maximum accumulated lethality was found under the free axial mode and the minimum in static. For each processing mode, the quality changes were found to correspond with the amount of lethality achieved.

Several retorting processes have investigated residual air and viscosity. One study compared static and reciprocation oscillations during the thermal treatment of flexible retort pouches (MacNaughton et al., 2018). The researchers discovered that agitated stirring resulted in higher heat penetration rates at all viscosities, with a reduction of up to 27% in processing time compared to static.

### 8.2 Heat transfer (heating)

The rate of heat transfer through a material can be influenced by the type of heating applied. Studies have compared different heating modes to determine their effect on the processing time and heating behavior of products (Zhu et al., 2022). For instance, Ramaswamy and Grabowski (1999) processed Pacific salmon using steam–air and water immersion in a still retort and compared the influence of container types and heating mediums on the heating behavior of the product. This study found that the type of heating medium had almost no effect on the processing time, and it only significantly influenced the heating rate index.

Similarly, Adepoju et al. (2017) investigated the heat penetration of milkfish during flexible poaching and compared steam–air processing and water immersion treatment on milkfish in flexible pouches. The steam–air method was found to have a shorter CUT and overall processing time than the water immersion process. However, the outcomes of these investigations are not always the same due to various reasons such as different types of retorts used and the type of containers used. For instance, Ramaswamy and Grabowski used two distinct retorts and semi‐rigid plastic containers in addition to cylindrical metal cans, while they made use of one retort that could switch between modes and flexible pouches. Additionally, the thinner flexible pouches allowed for quicker thermal transfer than the containers and cans due to their greater slenderness.

As the effects of agitation and heat transfer on food processing are discussed, it is necessary to consider a broader range of food preservation techniques. This comparison emphasizes the distinct characteristics and applications of each preservation technique (Table 3).

**TABLE 3:** Preservation techniques and comparison.

Preservation technique	Description	Food products	Conditions of process	Detailed mechanism	References
Pasteurization	Utilizes controlled heating to deactivate pathogenic microorganisms and enzymes, preserving sensory and nutritional quality	Pasteurized Milk, Fruit Juices (e.g., Orange Juice), Pasteurized Beer	Typically, 60–100°C; time and temperature dependent on product acidity and water activity	Low‐level thermal process targeting pathogenic bacteria in low‐acid (pH > 4.5) and high‐acid foods (pH ≤ 4.5)	Tavman et al. (2019)
Sterilization	Applies high‐temperature heat treatment to achieve complete inactivation of all forms of microorganisms, including spores	Canned Vegetables, Canned Meat, Shelf‐Stable Low‐Acid Foods	Exceeds 100°C, often 115–130°C; specific time–temperature combinations, e.g., 121.1°C for 3 min for a 12D reduction of *Clostridium botulinum*	Intensive thermal processing aimed at achieving commercial sterility in low‐acid foods	Ramesh (2020)
Refrigeration	Lowers the storage temperature to inhibit microbial growth and enzymatic activity, maintaining food quality for short‐term preservation	Chilled Fresh Produce, Dairy Products (e.g., Cheese), Chilled Raw Meat	Storage temperatures typically above freezing and below 15°C	Reduction of metabolic and microbiological activity through lower temperatures without freezing	Augusto et al. (2018)
Freezing	Transforms water in food to ice, significantly slowing down microbial activity and biochemical reactions	Frozen Vegetables, Frozen Dairy Products like Ice Cream, Frozen Meat Products	General practice requires temperature reduction to around −18°C for storage	Three‐stage process: Precooling to freezing point, phase transition with latent heat removal, and tempering to final storage temperature	Tavman and Yilmaz (2017)
Ohmic heating	Employs electric current to generate heat internally within the food product, enhancing quality and reducing processing time	Liquid Egg Products, Fruit Juices (e.g., Apple Juice), Soups with Particulates	Rapid volumetric heating with variable conditions based on food composition	Electrical resistance of food causes internal heating, effective for liquid–solid mixtures and viscous fluids	Rodrigues et al. (2021)
Microwave heating	Utilizes electromagnetic waves for rapid internal heating, suited for various food processing applications	Microwaveable Ready Meals, Snacks like Popcorn, Reheatable Food products	Utilizes specific microwave frequencies (915 or 2450 MHz), achieving rapid temperature increase	Electromagnetic waves cause dipole rotation and ionic polarization in food molecules, generating internal heat	Ahmed and Ramaswamy (2007)

## 9 FUTURE RESEARCH

### 9.1 Avenues of future research

During the review of the literature, some areas were noted to be lacking in scientific research. Verheyen et al. (2021) suggested that investigating the role of fat droplets' presence and fat content on heat transfer and microorganism destruction for reciprocating agitation retorts is necessary. Singh and Ramaswamy (2015) discussed conflicting evidence for can orientation heating in still retorts, with the majority of studies focusing on liquid products, indicating the need for further research. Ates et al. (2014) also highlighted the need for further study to analyze the efficacy of the reciprocation agitating technique.

### 9.2 Current state

As with any technology, innovation is constantly occurring. Retort processing began using still batch retorts but has evolved to include various heating, agitation, and rate methods. In the last 10 years, reciprocal agitation has been the focus of research. Of note, many of these retorts have been retrofitted to be able to process using newer methods, including reciprocal agitation. Researchers investigated the possibilities of modifying a static steam retort into a reciprocation agitation retort (Singh et al., 2015). This modification was found to be successful and created a 3–8 times reduction in equilibration time. The heat transfer coefficients also increased by 2–7 times.

### 9.3 Economic/environmental feasibility

As with any business, the financial feasibility of operations needs to be taken into consideration. It is essential to evaluate how retorting technology compares to cost when innovation occurs. In a study by Cacace et al. (2020), the expense of using high‐pressure processing (HPP), indirect thermal pasteurization, and retort thermal processing for orange juice was compared. Both thermal processing methods were found to be more cost‐effective than HPP in terms of all possible costs. The cost of processing 1 kg of juice using HPP was 1.78 times that of processing it with TP‐indirect treatment, and the cost was 1.40 times that of TP‐retort treatment. The most significant difference is in the start investment, where retort processing is the cheapest by a long margin but has a high operating cost when compared to indirect thermal treatment. HPP consumes approximately 2,700,000 kg, orange juice from TP‐indirect consumes 2,799,360 kg per year, and retort processing creates 2,640,000 kg per year. When the cost of production is taken into account, increased output may enable extra investment with a greater return. Gil et al. (2020) conducted an experimental study on a vertical retort and found that the venting stage accounted for a significant portion of the total energy consumption and affected the temperature distribution in the retort. The study recommended reducing the venting time and optimizing the venting pressure to improve the energy efficiency and thermal distribution of the process. Regarding the retort's environmental impact, a study by Cacace et al. (2020) found that it had the best scores in 13 of the 18 categories evaluated, including global warming, terrestrial ecotoxicity, and terrestrial acidification. However, it received the lowest score in terms of land use and mineral resource scarcity. In future research, methods to reduce environmental impact may be investigated.

### 9.4 Future of processing

Technological advancements and innovative research will characterize the future of retort processing. Chen et al. (2008) developed an online correction method for continuous retorts that improves temperature control accuracy significantly by utilizing a feedback control system for real‐time adjustments. This method is critical for food safety and quality, demonstrating how modern technology can improve traditional processes. The toroidal can, invented by van Droogenbroeck et al. (2021), is a new geometric form that can significantly reduce processing times by 40.2%. This innovation holds great promise for integrating into existing retort systems, providing a practical approach to commercial manufacturing efficiency improvements. Singh et al. (2018) investigated resonance‐acoustic mixing technology, presenting a novel method of agitating a can. This technique outperforms traditional rotary agitation by maintaining the same heat transfer rates and quality retention as reciprocating agitation. This advancement demonstrates the field's ongoing innovation. Fu et al. (2021) discovered that adding tea products to instant rice after sterilization can improve its flavor and texture, thus improving the quality of sterilized products. This discovery opens new avenues for post‐processing improvements. Calderón‐Alvarado et al. (2016) have significantly contributed to our understanding of natural convection in cylindrical cavities during sterilization. Their work involves viscosity modeling and is critical for ensuring food safety and product quality. Bassett et al. (2020) emphasized the advantages of reducing retort processing times. Their discovery that shortening the processing time from 90 to 60 min improves the quality of canned quick‐cooking dry beans is a significant step forward. This method improves product quality, processing efficiency, and production costs, making it highly beneficial to the food industry.

## 10 CONCLUSION

Thermal retort processing is a widely used and cost‐effective method of food preservation with various heating methods, including water immersion, crateless, and continuous retorting, each with its advantages and disadvantages. Ongoing research and development are aimed at improving the safety, quality, and efficiency of food preservation through new technologies such as toroidal cans and resonance‐acoustic mixing technology. The efficacy of microbial inactivation is a primary aim of thermal processing, and research is ongoing to improve it with actual food systems via reciprocal agitation. Heat transfer through a material can be altered by the type of heating applied, and studies have been conducted to compare different modes. Overall, thermal retort processing is a constantly evolving field with ongoing research and development aimed at improving the safety, quality, and efficiency of food preservation.

## AUTHOR CONTRIBUTIONS

**Paulina Simoneth Jimenez:** Data curation (equal); resources (equal); validation (equal); writing – original draft (equal). **Sneh Punia Bangar:** Formal analysis (equal); project administration (equal); visualization (equal); writing – original draft (equal); writing – review and editing (equal). **Mathew Suffern:** Data curation (equal); formal analysis (equal); investigation (equal); resources (equal); writing – original draft (equal). **William Scott Whiteside:** Formal analysis (equal); funding acquisition (equal); project administration (equal); supervision (equal); writing – review and editing (equal).

## FUNDING INFORMATION

No funding was provided for this research.

## CONFLICT OF INTEREST STATEMENT

The authors declare no conflict of interest relevant to this article.

## ETHICS STATEMENT

The present study did not include any procedures on humans or animals.

## Data Availability

The data that support the findings of this study are available from the corresponding author upon reasonable request.
